# Drug waste minimisation and cost-containment in Medical Oncology: Two-year results of a feasibility study

**DOI:** 10.1186/1472-6963-8-70

**Published:** 2008-04-01

**Authors:** Gianpiero Fasola, Marianna Aita, Luisa Marini, Alessandro Follador, Marina Tosolini, Laura Mattioni, Mauro Mansutti, Andrea Piga, Silvio Brusaferro, Giuseppe Aprile

**Affiliations:** 1 Department of Medical Oncology, University Hospital of Udine, 33100 Udine, Italy; 2 Unit of Pharmacy, University Hospital of Udine, 33100 Udine, Italy; 3 Department of Experimental and Clinical Pathology and Medicine, University Hospital of Udine, 33100 Udine, Italy

## Abstract

**Background:**

Cost-containment strategies are required to face the challenge of rising drug expenditures in Oncology. Drug wastage leads to economic loss, but little is known about the size of the problem in this field.

**Methods:**

Starting January 2005 we introduced a day-to-day monitoring of drug wastage and an accurate assessment of its costs. An internal protocol for waste minimisation was developed, consisting of four corrective measures: 1. A rational, per pathology distribution of chemotherapy sessions over the week. 2. The use of multi-dose vials. 3. A reasonable rounding of drug dosages. 4. The selection of the most convenient vial size, depending on drug unit pricing.

**Results:**

Baseline analysis focused on 29 drugs over one year. Considering their unit price and waste amount, a major impact on expense was found to be attributable to six drugs: cetuximab, docetaxel, gemcitabine, oxaliplatin, pemetrexed and trastuzumab. The economic loss due to their waste equaled 4.8% of the annual drug expenditure. After the study protocol was started, the expense due to unused drugs showed a meaningful 45% reduction throughout 2006.

**Conclusion:**

Our experience confirms the economic relevance of waste minimisation and may represent a feasible model in addressing this issue.

A centralised unit of drug processing, the availability of a computerised physician order entry system and an active involvement of the staff play a key role in allowing waste reduction and a consequent, substantial cost-saving.

## Background

The past ten years have seen a significant and progressive cost rising in Medical Oncology [1], largely due to the increase in cancer prevalence and the incorporation into clinical practice of novel, highly expensive drugs [2]. Indeed, the formidable bounce of recent scientific progress has led to the development, approval and licensing of novel, both cytotoxic and biological agents; these drugs have shown efficacy in clinical trials, provide further hope to cancer patients but are among the costliest in medical care [1].

The cost of one cycle of chemotherapy may range from 2,500 $ for docetaxel 75 mg/m2 to 4,000 $ for pemetrexed 500 mg/m2, both delivered every three weeks [3]. As for biological agents, one month of treatment costs from 2,300 $ for erlotinib to more than 4,000 $ for both trastumuzab and bevacizumab [1,4]. Even more expensive is cetuximab, that sells for 8,700 $ monthly [1].

Ims Health, provider of consulting services for pharmaceutical and healthcare industries, anticipates that by 2008 antineoplastic drugs will become the top therapeutic area and their market will total over 40 billions USD, an almost 50% increase as compared with 2004 [5].

Thus, the "oncologic time bomb" predicted in 1999 by an American Cancer Society task force has exploded [2] and the question of how patients and society will afford dramatically rising drug payments remains partly unanswered.

Several strategies have been suggested to this end: from the promotion of an evidence-based medicine to the adoption of validated endpoints both in clinical studies and in the process of drug approval [6]; from the search of relevant biomarkers for a better identification of responsive patients [7] to a proper allocation of health systems resources toward the fields of disease prevention and early detection [8]. Overall, it seems that comprehensive actions resulting from a thoughtful debate among the oncological community, the government, pharmaceutical industries and health insurers are needed to secure the financial future of health systems. Nonetheless, the formulation of satellite measures, short-run but also zero-cost may be highly desirable in such a scenario.

Drug waste may be defined as the consequence of an inappropriate disposal of unused or partially used ampoules, vials, or syringes of drugs [9]. It has been previously demonstrated that inefficiency of drug use and waste production may lead to a distinct economic loss, though experiences are limited and most studies are dated or focus on other therapeutic areas [9-12]. Decreasing waste is an attractive cost-cutting strategy since it neither limits specific drug use nor affects quality of care.

Our Department of Medical Oncology is a research-oriented academic unit, admitting about two thousands new patients every year. Facilities include an eighteen-bed day-hospital service and a fourteen-bed ward. The Clinic hosts a centralised unit for drug preparation and is equipped with a homegrown computerised physician order entry (CPOE) system; these features offer a unique opportunity for a sound management of drug preparation, prescription and administration.

In this framework, a project of drug use surveillance and waste reduction was designed and launched at the end of 2004. The project aimed at:

- monitoring the global amount of CT waste during a one-year observation period

- estimating the resulting economic loss and the relative influence of each drug

- measuring the cost-saving effect of a number of proposed corrective measures. More specifically, a *per pathology/per drug *organisation of treatment sessions over the week – in order to allow the re-use of leftovers while respecting drug stability – as well as a reasonable rounding of drug dosages (i.e., within 5% of calculated dose) [13] were encouraged. Multi-dose vials, that maintain much longer microbial and chemical stability, were used whenever possible, and – depending on drug unit pricing – the most convenient vial sizes were selected for use among available options.

Here we report on first two-year results.

## Methods

### 2005: observation phase

Since January 2005, the number of dilution cycles for the whole Hospital and for each Department was recorded monthly. Four dedicated laboratory technicians started a day-to-day monitoring of 29 prescription drug order forms, actual use and resulting waste. More specifically, a daily log was manually filled in by individual staff components of the cytotoxic reconstitution unit, who analysed the total amount of each drug prescription and the real amount of consumed drugs, and computed the difference. The Hospital Pharmacy provided a periodical report on the negotiated price per milligram of each observed drug, so that planned and *de facto *expense could be compared and the economic loss due to waste exactly determined.

Monthly variations in the waste of each drug and possible reasons were recorded. A Web literature search was performed and different domestic and international realities analysed for matching experiences.

Finally, an internal protocol for waste minimisation could be developed, consisting of four major corrective measures:

- a rational, *per pathology/per drug *distribution of chemotherapy sessions over the week, in order to allow the re-use of leftovers in other patients on the same or the following day, while respecting both chemical and microbiological drug stability. In particular, a model of organisation was devised and launched, scheduling the CT sessions for gastrointestinal malignancies to take place on Monday and Wednesday, those for thoracic malignancies on Tuesday, and breast cancer treatment sessions on Thursday and Friday

- the choice, whenever possible, of multi-dose vials, that maintain microbial and chemical stability for up to 28 days

- a reasonable rounding of drug dosages (i.e., within 5% of calculated dose) [13], to fit with available vial sizes/leftovers and avoid the waste of unstable medications

- the selection of the most convenient vial size among different available options, depending on drug unit pricing and on an accurate estimate of the daily actual need of each drug, based on the analysis of validated CPOE prescriptions

### 2006, first semester

Starting January 2006, the protocol of waste reduction was shared with all staff members and formally adopted. Monthly dilutions, as well as every-day drug prescription and actual consumption continued to be strictly recorded. Possible variations in drug unit pricing were documented.

Drug recovery and resulting money saving were registered monthly for each observed drug. Drug waste cost for the whole year was estimated on the basis of first semester's results and compared with 2005 expenses due to leftovers. Both figures were put in proportion and compared with effective 2005 and estimated 2006 total drug expenditures of the Department, respectively.

A final report was prepared at the end of this period and distributed to all members of the medical staff.

### 2006, last semester

Starting July 2006, the Authors decided to focus waste minimisation policies and economic analysis on the six drugs that – on the basis of 2005 observations and 2006 first semester's results – appeared to play a primary role from a cost-cutting perspective. In particular, cetuximab, docetaxel, gemcitabine, oxaliplatin, pemetrexed and trastuzumab were selected for analysis. Despite a relatively low drug waste cost during 2005, cetuximab was chosen for two reasons: first, its use was predicted to greatly increase during the following years, as a consequence of marketing approval in Italy in July 2005; second, it was one of the drugs for which a rational allotment of treatment sessions was expected to produce the maximum effect. Although its waste proportion and waste cost were relatively high, topotecan was excluded from further analysis since its occasional use made it a poor candidate for waste recovery. In the same way paclitaxel was not included among "hot drugs", since the introduction of multidose vials from the beginning of the year had allowed to avoid any further drug loss.

In October 2006, an effort was made to improve the practice of dose rounding; indeed, all staff members were provided with a leaflet indicating the most reasonable dose rounding depending on body surface/weight and available vial sizes. Analogous brochures were made available in all Day Hospital offices.

Projected waste cost of the six hot drugs for the whole year and its proportion relative to the overall pharmaceutical expenditure were calculated and compared with both 2005 findings and the initial results (i.e. those of first semester) of waste-containment policies. Finally, 2006 figures were taken all together and compared directly to 2005 observations.

A decrease in negotiated drug prices occurred in this period was taken into account when comparing waste costs of the first vs the last six months of the year; in particular, all estimates were repeated as if prices didn't show any variation. For the same reason, an average price per milligram was used when comparing 2005 vs 2006 figures.

## Results

### 2005: observation phase

Monthly mean dilutions were 1,102 for the whole Hospital, 633 for the Oncology Unit only.

Waste proportion for all 29 drugs equaled 9.6% of the total amount of reconstituted drugs, with a net loss of 180,000 €, corresponding to 6.4 per cent of the Department's annual drug expenditures (Table 1).

**Table 1:** 2005 baseline evaluation of drug request, drug consuming, waste proportion and correspondent cost

**Drug**	**Total drug prescribing (mg) **	**Total drug consuming (mg)**	**Waste proportion (%)**	**Negotiated^a ^drug unit pricing (€/mg)**	**Cost of consumed drugs (€)**	**Drug waste cost (€) **
**Bleomycin**	1735	1755	1.1	1.19	2088	24
**Carboplatin**	115367	123380	6.5	0.082	10117	657
**Cetuximab**	23110	24000	3.7	2.079	49896	1850
**Cyclophosphamide**	834568	943265	11.5	0.005	4716	543
**Cisplatin**	76300	83094	8.2	0.172	14292	1169
**Dacarbazine**	8310	10100	17.7	0.023	232	41
**Docetaxel**	31957	33640	5.0	7.525	253141	12665
**Doxorubicin**	24980	26380	5.3	1.46	38515	2044
**Liposomial Doxorubicin**	1406	1500	6.3	17.69	26535	1663
**Epirubicin**	64765	67160	3.6	1.907	128074	4567
**Etoposide**	63176	72300	12.6	0.022	1591	201
**5-fluorouracil**	5149000	5769000	10.7	0.002	11538	1240
**Fotemustine**	1653	2288	27.8	2.15	4919	1365
**Gemcitabine**	1692015	1790600	5.5	0.142	254265	13999
**Iphosphamide**	43150	52000	17.0	0.014	728	124
**Irinotecan**	95629	97690	2.1	1.76	171934	3627
**Methotrexate**	23531	26690	11.8	0.055	1468	174
**Mitomycin**	273	470	41.9	2.06	968	406
**Mitoxantrone**	134	150	10.7	5.39	808	86
**Oxaliplatin**	84095	90050	6.7	3.71	334085	22093
**Paclitaxel**	120948	126502	4.4	2.376	300569	13196
**Pemetrexed**	36290	42500	14.6	2.54	107950	15773
**Raltitrexed**	36	42	14.3	62.5	2625	375
**Topotecan**	192	392	51.1	64.72	25370	12944
**Trastuzumab**	65003	79270	17.9	4.69	371776	66912
**Vinblastine**	169	240	29.6	0.991	238	70
**Vincristine**	62	71	12.7	2.09	148	19
**Vindesine**	3	5	40.0	20.78	104	42
**Vinorelbine**	13659	14530	5.9	1.96	28479	1707
**TOTAL**	8571516	9479064	9.6		2147169	179576

^a^2006 official hospital drug prices in Italy are available at [21]

Fluctuations in waste proportion appeared to be of different magnitude for different drugs, and median wastage rate showed marked variability as well, ranging from 1.1% for bleomycin to more than 50% for topotecan.

**Figure 1:**
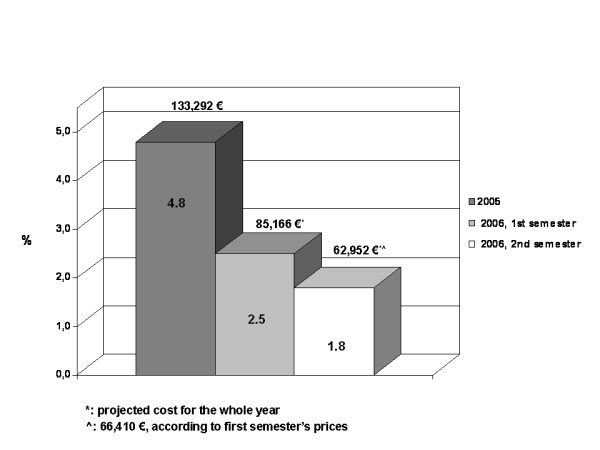
Waste cost proportion of "hot" drugs: 2005 vs 2006, first and second semester.

Waste cost of six compounds only, namely cetuximab, docetaxel, gemcitabine, oxaliplatin, pemetrexed and trastuzumab, accounted for 74% (= 133,292 €) of the total cost of wastes (Table 1) and 4.8 per cent of the Department's annual expenditures (Figure 1). On the other hand, their waste amount was 14% only of all the waste and their annual wastage rate was generally lower than 10%, with the only exception of pemetrexed and trastuzumab (wastage rate 14.6% and 18%, respectively).

### 2006, first semester

Median number of monthly dilutions was consistent with 2005 figures: 1,006 for the whole Hospital, 621 for the Oncology Department. Notably, no variation occurred in drug pricing nor in staff cost.

Following the application of waste containment measures, waste proportion for all drugs decreased from 9.6 to 6.5%, meaning a 41 per cent reduction of the overall waste cost as compared to the previous year and a decrease from 6.4 to 3.1% with respect to the estimated pharmaceutical expenditure (Table 2).

**Table 2:** 2006, first semester results

**Drug**	**Total drug prescribing (mg)**	**Total drug consuming (mg)**	**Waste proportion (%)**	**Negotiated^a ^drug unit pricing (€/mg)**	**Cost of consumed drugs (€)**	**Drug waste cost (€)**
**Bleomycin**	0	0	0	1.19	0	0
**Carboplatin**	62975	63185	0.3	0.082	5181	17
**Cetuximab**	47940	49300	2.8	2.079	102495	2827
**Cyclophosphamide**	334314	356500	6.2	0.005	1782	111
**Cisplatin**	40664	42190	3.6	0.172	7257	262
**Dacarbazine**	42730	45700	6.5	0.023	1051	68
**Docetaxel**	15630	16500	5.3	7.525	124162	6547
**Doxorubicin**	11532	11880	3	1.46	17345	508
**Liposomial Doxorubicin.**	960	1090	12	17.69	19282	2300
**Epirubicin**	31350	31580	0.7	1.907	60223	439
**Etoposide**	11855	14200	16.5	0.022	312	52
**5-fluorouracil**	2769077	3019370	8.3	0.002	6039	501
**Fotemustine**	1000	1040	3.8	2.15	2236	86
**Gemcitabine**	786330	802000	2	0.142	113884	2225
**Iphosphamide**	132050	137000	3.6	0.014	1918	69
**Irinotecan**	47995	48695	1.4	1.76	85703	1232
**Methotrexate**	9118	9700	6	0.055	533	32
**Mitomycin**	196.5	250	21.4	2.06	515	110
**Mitoxantrone**	15	20	25	5.39	108	27
**Oxaliplatin**	47033	49000	4	3.71	181790	7298
**Paclitaxel**	43932	43932	0	2.376	104382	0
**Pemetrexed**	26020	30000	13.3	2.54	76200	10109
**Raltitrexed**	37	42	11.9	62.5	2625	312
**Topotecan**	114	168	32.2	64.72	10873	3495
**Trastuzumab**	38433	41328	7	4.69	193828	13577
**Vinblastine**	25	20	25	0.991	20	5
**Vincristine**	20	22	9	2.09	46	4
**Vindesine**	50.5	65	22.3	20.78	1351	301
**Vinorelbine**	6718	6785	0.9	1.96	13299	131
**TOTAL**	4508114	4821562	6.5		1134440	52645

^a^2006 official hospital drug prices in Italy are available at [21]

**Figure 2:**
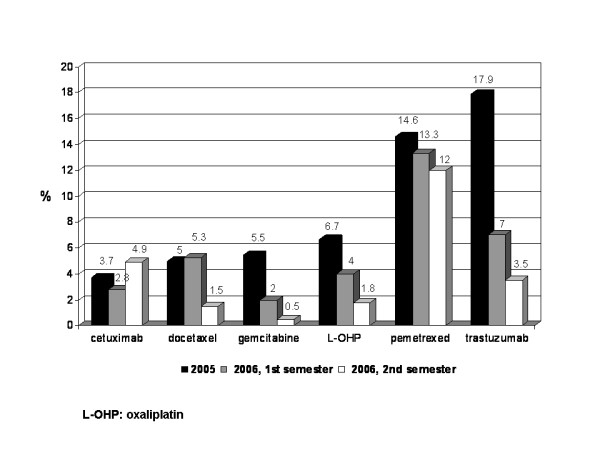
"Hot" drug decrease in waste proportion: 2005 vs 2006, first and second semester.

Corrective measures were successful in reducing the waste proportion of all "hot" drugs, with the only exception of docetaxel, whose leftovers did not show any substantial variation. In particular drug waste for gemcitabine, oxaliplatin and trastuzumab dropped from 5.5 to 2%, 6.7 to 4% and 17.9 to 7%, respectively (Figure 2).

The cost of wastage of the six "hot" compounds decreased from 4.8 to 2.5% of the overall pharmaceutical expenditure (Figure 1). Indeed, the projected cost of their leftovers for the whole year was estimated to be 85,166 € – as compared to 133,292 € in 2005, meaning a planned saving of about 50,000 €.

### 2006, last semester

The last semester of 2006 did not show any substantial variation in drug preparation time and in staff monthly workload: median number of dilutions was 1,159 for the whole Hospital, 710 for the Oncology Unit only. Staff cost was the same as the previous six months.

Starting July, 2006, waste minimisation policies and economic analysis were focused on the six drugs listed in the Methods section. By year's end, the positive trend in waste reduction was confirmed, with regard especially to the wastage of gemcitabine, docetaxel, oxaliplatin and trastuzumab, that showed a 75 (from 2 to 0.5%), 72 (from 5.3 to 1.5), 55 (from 4 to 1.8%) and 50 (from 7 to 3.5%) per cent further drop, as compared to the first six months (Figure 2). The decrease in the waste rate of pemetrexed was less important, from 13.3 to 12%, while cetuximab inverted its trend, with an increase in wastage rate from 2.8 to 4.9%. Nonetheless the overall projected waste cost further abated from 85,166 to 62,952 € (Figure 1), meaning a 26% absolute reduction and a 28% reduction of its fraction relative to the pharmaceutical global expenditure, from 2.5 to 1.8% (Figure 1).

The decrease in negotiated drug prices occurred in this period ranged from a minimum of 3% for trastuzumab to a maximum of 10% for pemetrexed. When repeating all estimates as if prices had not shown any variation (Table 3) we found that drug waste cost for cetixumab, docetaxel, gencitabine, pemetrexed and trastuzumab would have grown of 112, 94, 56, 1,130 and 364 euros, respectively, whereas that of oxaliplatin would have decreased, from 3,329 to 3,302 €. Overall, the waste cost of the six drugs would have raised of 1,729 € only, meaning a 24% decrease of its fraction of the overall drug expense, from 2.5 to 1.9%.

**Table 3:** 2006, second semester results

**Drug**	**Total drug prescribing (mg)**	**Total drug consuming (mg)**	**Waste proportion (%)**	**Negotiated^a ^drug unit pricing (€/mg)**	**Cost of consumed drugs (€)**	**Drug waste cost (€)**	**Drug waste cost according to 1st semester's prices (€)**
**Cetuximab**	31483	33100	4.9	2.01	66531	3250	3362
**Docetaxel**	20491	20811	1.5	7.23	150463	2314	2408
**Gemcitabine**	1132387	1138600	0.5	0.133	151434	826	882
**Oxaliplatin**	49370	50260	1.8	3.74	187972	3329	3302
**Pemetrexed**	31656	36000	12	2.28	82080	9904	11034
**Trastuzumab**	71345	73950	3.5	4.55	336472	11853	12217
**TOTAL**	1336732	1352721	1.2		974952	31476	33205

^a^2006 official hospital drug prices in Italy are available at [21]

## Discussion

In days when mankind's knowledge of cancer is greater than ever before [14] and the number and cost of new anticancer drugs are rising to unexpected heights, overcoming the disproportion between health needs and available resources represents a moral as well as an economic challenge [15].

Unfortunately, most of the strategies that have been proposed to escape the need for health care rationing [16] represent medium-long term solutions, whose impact on high and rising costs is expected to be appreciable in a hardly predictable future. Prompter suggestions are probably needed to control the problem in a short term-oriented manner.

In this perspective, our experience shows how a relatively simple policy of drug waste reduction may significantly decrease their cost impact on the overall pharmaceutical expenditure and allow a substantial cost-saving.

In 2005 a net loss of 180,000 €, corresponding to 6.4 per cent of the Department's annual expenditures (i.e. 2,800,000 €), could be attributed to futile drug leftovers. Waste cost of six high-priced and/or widely used drugs, i.e. cetuximab, docetaxel, gemcitabine, oxaliplatin, pemetrexed and trastuzumab, accounted for three-quarters of this loss and to 4.8 per cent of the Department's annual drug expenditures. A strict monitoring of drug use endorsed to acknowledge: first, that recovery policies would probably not apply to drugs showing minor fluctuations in monthly wastage rates, since low variability implied the existence of a somehow physiologic, thus unrecoverable, loss; second, that main reasons for drug waste were essentially the limited extent of CT medication shelf-life and the narrow availability of a range of vial sizes flexibly matching with possible drug dosages. Adopted corrective measures were the logical consequence of these findings: if drug instability is a basis for drug waste, it is reasonable to use, whenever possible, multi-dose vials, that retain a much longer microbial and chemical stability; and to operate a per pathology/per drug distribution system of chemotherapy sessions over the week, in order to allow the re-use of leftovers in other patients, while respecting drug stability. In the same way, if market available vial sizes are relatively few, it makes sense to round down the drug dose to the closest accessible vial size. Notably, dose-rounding has been considered acceptable to within 5% of calculated dose, since on the basis of pharmacokinetic and clinical issues this dose adjustment is not expected to have any significant effect on either response or toxicity [13,17-19].

In our work we show how the application of straightforward measures allowed to abate the waste amount of the most expensive antineoplastic drugs, its cost and its proportion of total drug expenditure. In particular, a direct comparison of 2005 figures with 2006 reveals how the overall waste amount for the six "hot" dugs dropped of 66%, its cost decreased of 45% (from 133,292 € to 73,975 €, using an average price per milligram) and its fraction of pharmaceutical global expenditure diminished from 4.8 to 2.2%.

Further expenditure cuts may hopefully emerge from other equally feasible solutions. The establishment of an ad hoc policy for recovering unused, unexpired oral antineoplastic drugs (including biological agents) would allow a considerable medication return and money-saving. A concrete cooperation with manufacturing companies should be solicited focusing on ways to improve and validate the stability of drugs, particularly those items that need to be used within hours, and on the production of more suitable final dosage forms (for example, the decrease in pemetrexed wastage rate was less important than for the other hot drugs, since the possibility of drug recovery suffered from the limits imposed by commercially available vial sizes).

Our study has some limitations. First, it was conducted at a single centre and its generalisability to settings of different size and with a potentially different mix of diagnoses and disease severity needs to be confirmed.

Second, the rigorous planning of chemotherapy sessions across the week – which plays a key role in waste containment – requires a strictly organised structure and such a policy may be difficult to pursue, especially in the absence of a clinical information system. The increase in cetuximab wastage rate during the second semester of 2006, which was probably due to a demanding 24-hour concentration of chemotherapy sessions for colorectal cancer patients, is an example of such a hindrance. At present, we are working toward the validation and inward testing of drug microbial stability, with the aim of easing the planning and escaping from the needs of a too rigid system.

Finally, it has been previously estimated that potential savings from the reduction of inefficiencies fall short of administration's cost containment [20]; thus, our strategy may best function as an interim measure, in hold of more comprehensive, long-term plans to achieve sustainable outcomes.

## Conclusion

Our study demonstrates that in Medical Oncology drug waste reduction is feasible and economically convenient. The existence of a centralised unit for drug manufacturing, providing a continuous surveillance on drug prescription and utilisation, a meticulous planning of daily workload, granted by CPOE, and an actively shared information and feedback among staff members are key elements to successfully pursue the proposed strategies.

The concept of "sustainability" should not allude only to the mandate of reducing health care expenditure. A sustainable oncology is economically affordable; at the same time it provides to all the community an equal right to proper levels of physical and mental well-being and aims to ceaseless progress and innovation. In this sense, the identification of easily applicable solutions, that allow to control rising costs while maintaining or improving the quality of patient care, remains challenging but highly attractive.

## Abbreviations

NSCLC: non-small cell lung cancer; i.e.: id est; CT: chemotherapy; USD: United States dollars; CPOE: computerised physician order entry; vs: versus

## Competing interests

The author(s) declare that they have no competing interests.

## Authors' contributions

All authors have substantially contributed to the research. Specifically GF, GA and LM conceived the idea for the study, and, with MA, designed and planned the research. All authors were involved in data collection; MA, LM, AF, MT, LM and MM analysed them. MA wrote the first draft of the manuscript; GF, LM, AP, SB and GA critically revised it. All authors read and approved the final version of the manuscript.

## Pre-publication history

The pre-publication history for this paper can be accessed here:


